# Supplementary material for: Using Deep Convolutional Neural Networks for Enhanced Ultrasonographic Image Diagnosis of Differentiated Thyroid Cancer
Source: Biomedicines. 2021 Nov 26;9(12):1771. doi: 10.3390/biomedicines9121771 (PMC8698578; doi:10.3390/biomedicines9121771)
Supplement: Supplementary file 1 [file biomedicines-09-01771-s001.zip › biomedicines-1447094-supplementary.pdf]

biomedicines

MDPI

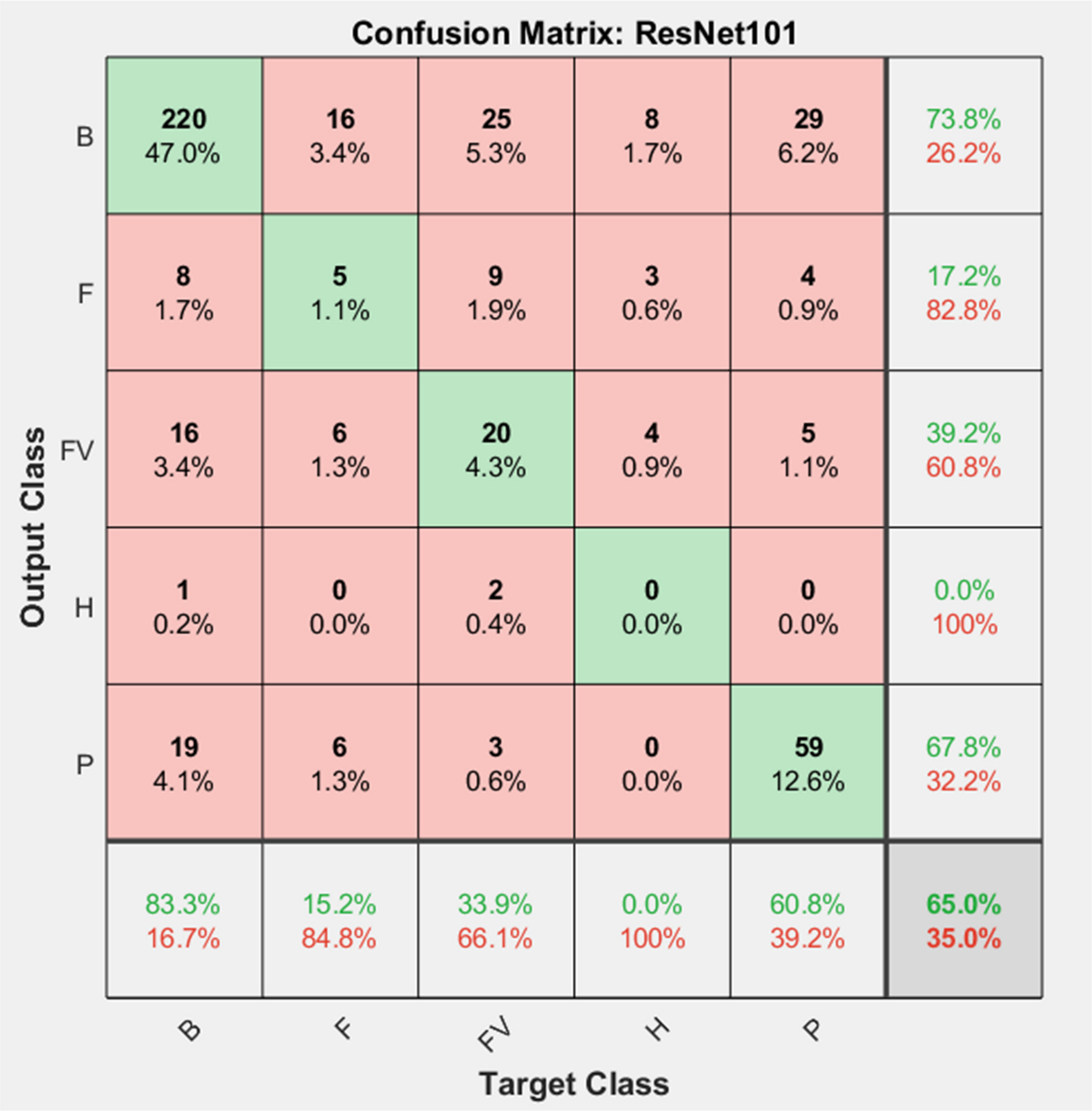

Figure S1. Confusion matrix of multi-class classification using ResNet101 in test set. B: Benign, F: Follicular thyroid carcinoma, FV: Follicular variant of papillary thyroid carcinoma, H: Hürthle cell carcinoma, P: Papillary thyroid carcinoma.
